# Anti-Inflammatory Effects of Melatonin and 5-Methoxytryptophol on Lipopolysaccharide-Induced Acute Pulpitis in Rats

**DOI:** 10.1155/2021/8884041

**Published:** 2021-02-12

**Authors:** Fatma Kermeoğlu, Umut Aksoy, Abdullah Sebai, Gökçe Savtekin, Hanife Özkayalar, Serkan Sayıner, Ahmet Özer Şehirli

**Affiliations:** ^1^Faculty of Dentistry, Department of Endodontics, Near East University, Nicosia, Mersin 10, Turkey; ^2^Faculty of Dentistry, Department of Maxillofacial Surgery, Cyprus Health and Social Science University, Omorfo, Mersin 10, Turkey; ^3^Faculty of Medicine, Department of Pathology, Near East University, Nicosia, Mersin 10, Turkey; ^4^Faculty of Veterinary Medicine, Department of Biochemistry, Near East University, Nicosia, Mersin 10, Turkey; ^5^Faculty of Dentistry, Departments of Pharmacology, Near East University, Nicosia, Mersin 10, Turkey

## Abstract

**Aim:**

The aim of this study was to investigate the possible therapeutic impacts of two pineal hormones, melatonin and 5-methoxytryptophol (5-MTX), in a rat model of acute pulpitis by analyzing biochemical and histopathological parameters.

**Methods:**

This research was done using 32 male and female Wistar albino rats with weight between 200 and 250 g. The rats were randomly divided into four groups: a control group (rats without any treatment), acute pulpitis (AP) group, AP+melatonin group, and AP+5-MTX group. In the AP-induced groups, the crowns of the upper left incisors were removed horizontally. Lipopolysaccharide solution was applied to the exposed pulp tissue before the canal orifices were sealed with a temporary filling material. Melatonin (10 mg/kg) and 5-MTX (5 mg/kg) were administered intraperitoneally. The rats were sacrificed 24 hours after pulp injury, and trunk blood and pulp samples were collected. The concentrations of TNF-*α*, IL-1*β*, MMP-1, and MMP-2 in sera and pulp samples were determined using ELISA assay kits.

**Results:**

TNF-*α*, IL-1*β*, MMP-1, and MMP-2 levels in the serum and pulp tissues were considerably higher in the AP group than the control group (*p* < 0.01‐0.001). In the AP+melatonin and AP+5-MTX groups, TNF-*α*, IL-1*β*, MMP-1, and MMP-2 levels in the serum and pulp tissues were significantly lower than in the AP group (*p* < 0.05‐0.001).

**Conclusions:**

Both melatonin and 5-MTX provided protective effects on acute pulpitis, which indicates they may be promising as a therapeutic strategy for oral disease.

## 1. Introduction

Pulpitis is generally associated with dental caries that lead to bacterial invasion of the dentinal tubules. Bacterial invasion induces host defense reactions in the dental pulp, including inflammatory and immune events. The goal of this reaction is to limit or prevent the progression of injury or damage of the pulp tissue [1]. The host defense response triggers a significant complex process involving various factors and elements, such as cytokines and matrix metalloproteinases (MMPs). During the whole process, inflammatory and immune events help to destroy bacteria and their by-products, but they also damage the pulp tissue at the same time because the process does not discriminate between enemies and the host tissue [2]. Consequently, in untreated cases, the condition leads to irreversible pulpitis with the formation of abscess in the pulp tissue and increased clinical symptoms.

One of the important inflammatory cytokines is tumor necrosis factor-*α* (TNF-*α*), which manages the coordination of the early host response to injury. Furthermore, it plays a central role between the initial stage and later inflammatory phases. Thus, TNF-*α* is often referred to as a proinflammatory cytokine [3]. Interleukin-1*β* (IL-1*β*) is another proinflammatory cytokine that takes part in mediating the inflammatory response. It modulates cell migration and the release of other mediators during the early stage of the inflammatory process [4]. MMPs are essential proteolytic enzymes that take part in several physiological and pathological processes, including inflammation [5]. MMPs can degrade almost all types of extracellular matrix components and play a crucial part in the tissue destruction of inflamed pulp. Recent studies have shown that MMPs take part in pulpitis, and the levels of MMP-1 and MMP-2 are considerably higher in pathological pulp tissue than in healthy pulp tissue [6–8].

Melatonin (N-acetyl-5-methoxytryptamine), a neuroendocrine hormone, is involved in various functions, such as reproduction, immunity, regulation of circadian rhythms, sexual development, and aging [9]. Melatonin also inhibits the matrix metalloproteinase activity, thereby inhibiting the production of proinflammatory cytokines, including IL-1*β* and TNF-*α* [10–13]. Another neuroendocrine hormone, 5-methoxytryptophol (5-MTX), is involved in several aspects of physiology and neuroendocrine roles. Like melatonin, 5-MTX also coordinates the biological rhythms and sexual behavior of various mammalian species, including humans. In addition to these, 5-MTX has significant biological functions as an antioxidant, immunomodulator, and anticancer agent [14].

The molecular structures of 5-MTX and melatonin are similar. However, they have an adverse effect on the light-dark cycle due to the presence of different functional groups. The secretion of melatonin increases after the onset of darkness and decreases significantly in the light phase, whereas 5-MTX increases during the light phase [15]. Recent studies have focused on the differences and levels of the effects of melatonin and 5-MTX [14, 16].

Several studies have looked at the effects of antioxidant and anti-inflammatory agents in apical and marginal periodontitis [17–23]. However, little is known regarding their effects on pulpitis. As far as we know, there is only one such study on melatonin, and there are none on 5-MTX [13]. Therefore, the aim of the present study is to examine the possible therapeutic impacts of melatonin and 5-MTX in a rat model of acute pulpitis by analyzing biochemical and histopathological parameters. The null hypothesis is that the groups do not differ significantly with respect to the levels of different inflammatory mediators and MMPs.

## 2. Material and Methods

### 2.1. Animals

Approval for the study protocol was obtained from the local animal ethics committee (protocol no: 2-16/2). This research was done with 32 male and female Wistar albino rats with weight between 200 and 250 g. All rats were housed in a temperature-controlled room (22 ± 2°C) with a 12-hour light/dark cycle. The rats had unlimited access to food and water.

### 2.2. Experimental Pulpitis Model

The rats were divided into four groups: a control group (rats without any treatment), acute pulpitis (AP) group, AP+melatonin group, and AP+5-MTX group. In the AP-induced groups, the rats were anesthetized by an intraperitoneal injection with a mixture of ketamine (100 mg/kg) and xylazine (10 mg/kg) and fixed in a supine position for surgery. The crowns of the upper left incisors were removed horizontally with a high-speed handpiece and round bur under water cooling.

Five microliters of lipopolysaccharide (LPS) from *Escherichia coli* O111:B4 (Sigma Chemical Co., St. Louis, MO, USA) was diluted in phosphate-buffered saline at a final concentration of 10 mg/mL. The LPS solution was applied to the exposed pulp tissue before the canal orifices were sealed with a temporary filling material. In the AP+melatonin and AP+5-MTX groups, melatonin (10 mg/kg, Sigma, St. Louis, MO) and 5-MTX (5 mg/kg, Sigma, St. Louis, MO) were administered intraperitoneally. The rats were sacrificed at 24 hours after the pulp injury, and trunk blood samples were collected. The intact incisor teeth in the control group and operated teeth in the AP groups were extracted, and pulp samples were also collected.

Blood samples were drawn into serum separator tubes. Sera were separated following centrifugation at 1500 *g* for 10 min and stored at -20°C until analysis. The blood from the exposed open root apex of the extracted incisors was collected by a sterile cotton pellet. The pellet was held at the root apex for 45 s to ensure the absorption of the pulpal blood. Afterwards, the pellets were treated as previously described [24]. Pellets were put in 1 mL of saline in heparin-coated tubes. The samples were kept at a temperature of 20°C until analysis. The samples were thawed and centrifuged twice at 12,000 *g* for 10 min at a temperature of 4°C.

### 2.3. Biochemical Analysis

Concentrations of TNF-*α*, IL-1*β*, MMP-1, and MMP-2 in sera and pulp samples were determined by commercially available rat-specific ELISA assay kits (Rat TNF-alpha Platinum ELISA, BMS622, Bender MedSystems GmbH, Vienna, Austria; Rat IL-1beta Platinum ELISA, BMS630, Bender MedSystems GmbH, Vienna, Austria; Rat MMP-1 ELISA Kit, E-EL-R0617, Elabscience, Wuhan, China; and Rat MMP-2 ELISA Kit, E-EL-R0618, Elabscience, Wuhan, China). Assays were performed in the Animal Hospital Diagnostic Laboratory of Near East University. The ELISA tests were carried out by following the directions of the manufacturers.

The washing steps of assays were done using an automated microtiter washer (MW-12A microplate washer, Mindray, Shenzhen, China), and absorbances were obtained using a microtiter plate reader at 450 nm (MR-96A microplate reader, Mindray, Shenzhen, China). TNF-*α* and IL-1*β* concentrations in the blood samples were expressed as pg/mL, and MMP-1 and MMP-2 concentrations were expressed as ng/mL. TNF-*α* and IL-1*β* concentrations in the pulp samples were expressed as pg/mg total protein, and MMP-1 and MMP-2 concentrations were expressed as ng/mg total protein.

The Bradford protein determination method was used for protein quantitation in the pulp samples [25]. Five microliters of each pulp samples was dispensed into the wells of microtiter plates, and then 250 *μ*L of Bradford reagent (B6916, Sigma-Aldrich, MO, USA) was added and mixed for 30 seconds. Samples were incubated at room temperature for 30 minutes, and then the absorbance was measured at 595 nm. BSA (bovine serum albumin, P0914, Sigma-Aldrich, MO, USA) was used as a standard, and all samples were assayed in duplicate.

### 2.4. Histopathological Analysis

Surgically removed teeth were dissected and fixed in 10% neutral-buffered formalin solution. The samples were processed in the Medical Pathology Laboratory of the University. The teeth samples were demineralized using 5% nitric acid (pH 7.4) and embedded in paraffin. The paraffin blocks that contained the teeth samples were serially sectioned with an average thickness of 4-5 micrometers. Histopathological analyses were conducted on hematoxylin-eosin-stained samples using the bright field mode of a light microscope (Zeiss-Axio Scope A1, Carl Zeiss, Gottingen, Germany).

**Figure 1:**
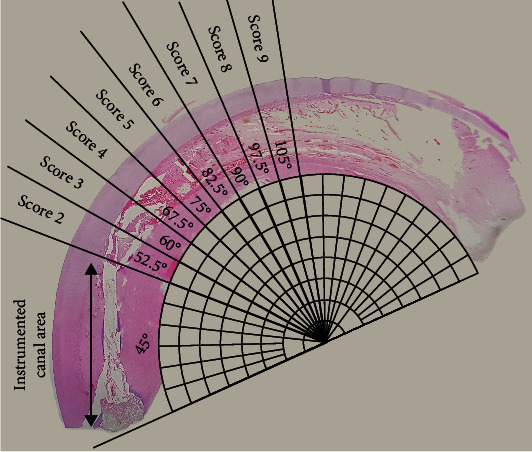
The histomorphologic parameters were classified according to a standardized scoring system based on a grading scale. A half circle was placed tangentially to the inner curve of the tooth arch. The uninstrumented root canal areas were divided into equal angular regions (45 to 105 degrees) which forming the scoring system.

Two histological slides (4 fields each) were assessed for each tooth. The histomorphologic parameters were the extension of the inflammatory reaction, the intensity of the inflammatory infiltrate, and extension of pulp necrosis, which were classified according to a standardized scoring system (Table 1). A half circle was placed tangentially to the inner curve of the tooth arch. The uninstrumented root canal areas were divided into equal angular regions (45 to 105 degrees) to form the scoring system, as shown in Figure 1. The specimens were examined, and each parameter was scored 1 to 9 (with 1 being the best result and 9 being the most severe) for the extension of the inflammatory reaction. The intensity of the inflammatory infiltrate was scored 1 to 4 on a scale from none or a few scattered inflammatory cells present in the pulp area corresponding to the pulp exposure, to the severe inflammatory cell infiltrate involving the coronal pulp or abscess present. The specimens were also scored for the presence or absence of the inflammatory edema. Extension of pulp necrosis on the 45 to 105 degrees area (Figure 1) was evaluated and scored on a scale from absence to the more than 30% existence of the related area. Evaluations were performed by an experienced pathologist who was blinded and calibrated to the experimental groups.

### 2.5. Statistical Analysis

Statistical analyses were carried out using GraphPad Prism 7.0 (GraphPad Software, San Diego, CA, USA). All results are expressed as the means ± SD. A one-way analysis of variance (ANOVA) was used for comparison of the levels of TNF-*α*, IL-1*β*, MMP-1, and MMP-2 in the serum and pulp tissues. Tukey's test was used for further analysis for binary comparisons. *p* values below 0.05 were regarded as significant.

## 3. Results

**Figure 2:**
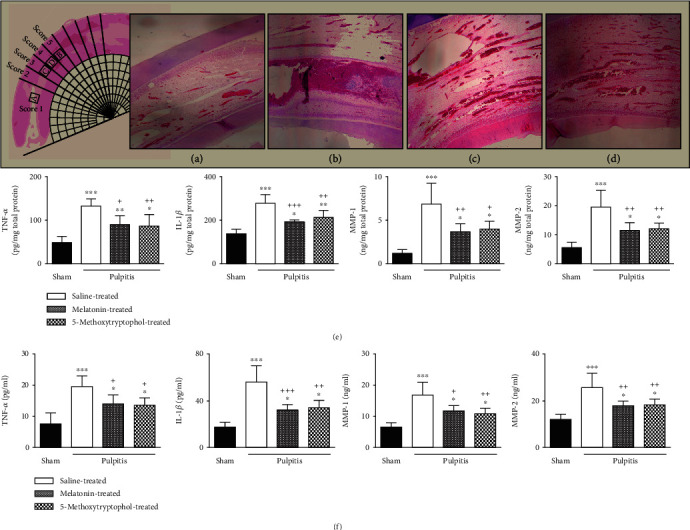
Melatonin and 5-MTX inhibited proinflammatory cytokine and MMP levels in the serum and pulp tissue in acute pulpitis. Hematoxylin-eosin-stained samples of pulp from the control, AP, AP+melatonin, and AP+5-MTX groups (a–d). Increased proinflammatory cytokine levels in the serum and pulp were observed at the saline-treated AP group. Abdominal injection of melatonin and 5-MTX ameliorated the increased level of TNF-*α*, IL-1*β*, MMP-1, and MMP-2 in the serum and pulp tissue of acute pulpitis-induced rats (e, f).

TNF-*α*, IL-1*β*, MMP-1, and MMP-2 levels in the serum and pulp tissues (Figure 2) were considerably higher in the AP group than the control group (*p* < 0.01‐0.001). In contrast, in the AP+melatonin and AP+5-MTX groups, these levels decreased significantly in comparison with the AP group (*p* < 0.05‐0.001). The quantification of histopathologic findings is shown in Table 2. Intensive neutrophil infiltration was observed in the AP group 24 hours after the dental pulp was exposed to the oral environment. At 24 hours, inflammatory reactions including inflammatory edema and pulp necrosis were observed in all of the samples, with scores being statistically different from samples from the control group (*p* < 0.05). There was considerable improvement in the AP+melatonin and AP+5-MTX groups compared to the AP group; however, when the two treatment groups were compared with each other, no statistically significant difference was present regarding histomorphologic scores (*p* > 0.05) (Table 2).

## 4. Discussion

The goal of this study was to investigate the anti-inflammatory potential of two different pineal gland hormones in a rat model of pulpitis in its acute stage. We have demonstrated that the systemic administration of melatonin and 5-MTX was able to modulate acute inflammation by inhibiting the increase of proinflammatory cytokines and proteolytic enzymes. Previous studies have demonstrated that melatonin reduces the degree of tissue injury in various animal models of systemic or local inflammation, as well as in clinical trials with human subjects [26]. Melatonin and 5-MTX are water-soluble, cell-permeating, nontoxic compounds with strong antioxidant properties. They can also diffuse through many biological membranes, including the blood-brain barrier [15]. Therefore, we wondered whether the systemic administrations of these compounds might be effective in suppressing the inflammatory process established in a rat model of acute pulpitis.

Previous studies have shown that it is possible to reproducibly induce pulpal inflammation in small rodents by different approaches, including the insertion of soft carious dentine, lipopolysaccharide stimulation, or pulpal exposure [27–30]. In the present study, acute pulpitis was experimentally induced by the topical application of LPS for 24 hours following pulpal exposure of the maxillary incisors. Since the rodent incisors represent continuously erupting teeth with high healing capability following pulp injury, they have been used to assess variations in biological factors in acute pulpal inflammation in several studies [5, 31, 32].

LPS is among the most potent activators of the human innate immune system. Many cytokines (including TNF-*α* and IL-1*β*) and MMPs (such as MMP-1 and MMP-2) are induced in response to LPS from human peripheral blood mononuclear cells, dental pulp cells, and gingival fibroblasts [33]. LPS-induced dental pulp inflammation represents a stable experimental inflammation model for the dynamic observation of the progress of acute and chronic pulpitis, as well as other oral inflammatory diseases in rodents [27, 34]. Previous studies have shown that LPS-treated inflammation is induced in a time- and dose-dependent manner in dental pulp cells [35–37].

Renard et al. [38] demonstrated that the inflammatory/immune response of pulp injury with LPS is quicker and stronger than that without LPS. Huang et al. [39] also showed that LPS-induced dental pulp inflammation changes dynamically between 6 and 24 hours in rats. Li et al. [13] indicate that the most severe inflammation in acute pulpitis occurs at 24 hours postsurgery in rats. Consistent with the literature, the duration of the current experiment was designed for 24 hours. Our findings demonstrated that LPS was capable of provoking acute pulpitis successfully within 24 hours.

Macrophages produce proinflammatory cytokines, such as IL-1*β* and TNF-*α*, in response to LPS in the cell walls of Gram-negative bacteria [37]. The expression levels of TNF-*α* and IL-1*β* validated the presence of inflammation in the pulp tissue [40]. IL-1*β* is considered to be a key mediator that promotes a variety of innate immune processes and exacerbates damage during chronic disease and acute tissue injury [4, 37, 41]. TNF-*α* also induces the activation of inflammatory cells, further increases the inflammatory response, and increases MMP-1 expression in pulp tissue [42].

Melatonin treatment decreases inflammation as a result of inhibiting the production of proinflammatory cytokines [12, 43, 44]. Previous studies have shown that the gene expression levels of TNF-*α* and IL-1*β* fluctuate between time points but reach their peak at 24 hours [40]. Yu et al. [37] stated that the gene expression levels of TNF-*α* and IL-1*β* decreased considerably when rats with acute pulpitis were treated with melatonin at one-day post-AP establishment. They also indicated that pretreatment with melatonin significantly attenuated the LPS-induced upregulation of proinflammatory cytokine gene expression [37]. In accordance with previous data, our findings revealed that TNF-*α*, IL-1*β*, MMP-1, and MMP-2 levels in the serum and pulp tissues were significantly higher in the AP group than the control group. However, in the AP+melatonin and AP+5-MTX groups, these levels decreased significantly compared to the AP group.

5-MTX is involved in physiological and neuroendocrine functions, but its cellular and molecular mechanisms have not been elucidated yet. Since it assists with the removal of inflammatory mediators, it bears similar characteristics to melatonin [15]. However, there are not enough studies in the literature on the effects of 5-MTX on TNF-*α* and IL-1*β*. Our findings showed that the inhibitory effects of melatonin and 5-MTX on proinflammatory cytokines lead to tissue-protective effects. These results are in agreement with Savtekin et al. [16], who stated that 5-MTX exhibits protective features by influencing MMP-2, TNF-*α*, and IL-1*β* levels in zymosan-induced synovial inflammation.

Moreover, since the inverse daily rhythms of melatonin and 5-MTX have been established, Lissoni et al. [45] demonstrated that these two indoleamines acting at various times of the day must be used together to derive benefits from immunomodulatory impacts. These findings raise intriguing questions regarding whether the simultaneous administration of melatonin during the dark period and 5-MTX during the light period of the day could be more effective. Thus, further work is required to establish the combined effects of these hormones.

MMPs are a family of calcium-dependent zinc-containing endopeptidases that consist of five subgroups: collagenases, stromelysins, gelatinases, membrane-type MMPs, and others [46]. They are expressed in epithelial, mesenchymal, and hematopoietic cells [47]. Experimental and clinical studies have shown that MMP-1 (a member of collagenases) and MMP-2 (gelatinase A) in particular modulate inflammation [48]. Our study has shown that the activation of proteolytic enzymes MMP-1 and MMP-2 is increased in acute experimental pulpitis induced by LPS. Therefore, these MMPs show activity by destroying extracellular matrix proteins by separating specific peptide bonds in different cells and tissues and play an essential role in pathological conditions [49].

Our results are consistent with the literature. In the melatonin and 5-MTX groups, the LPS application resulted in increased MMP-1 and MMP-2 activation in the serum and pulp tissue in comparison with the control group. The effect of melatonin on the expression levels of MMP-1 and MMP-2 has been studied in several models of inflammation [10, 11]. The antioxidant and anti-inflammatory properties of melatonin and 5-MTX in arthritis models have demonstrated that the protective effect is created by reducing the expression of MMP-1 and MMP-2 [16]. However, the effects of melatonin and 5-MTX on proteolytic enzymes have not been previously studied in pulpitis models. Thus, our study is an initial step and opens the gate for subsequent studies.

In this study, we indicated the role of 5-MTX in inhibiting pulpal inflammation for the first time. We compared the impacts of 5-MTX and melatonin on proinflammatory cytokines. An abdominal injection of 5-MTX and melatonin can significantly decrease the expression levels of TNF-*α*, IL-1*β*, MMP-1, and MMP-2 and can have a protective effect in acute pulpitis. This demonstrates that 5-MTX and melatonin may be promising therapeutic agents for oral diseases.

## 5. Conclusions

Our experimental study has revealed that proinflammatory cytokines and proteolytic enzymes were increased, and changes in biochemical parameters were accompanied by an increase in the serum and pulp tissue, indicating tissue damage. Melatonin and 5-MTX treatment were shown to reduce this damage significantly. As far as we know, this research is the first to investigate the anti-inflammation impacts of two pineal hormones in pulp inflammation. Our results suggested that melatonin and 5-MTX play an essential role in pulp tissue inflammation repair. Thus, more extensive and comparative clinical and experimental research is required for these agents to be used in clinics.

## Figures and Tables

**Table 1:** Parameters and scores used for evaluation.

Parameters	Scores
Extension of the inflammatory reaction	Score 1—absent
	Score 2—restricted to the 45 to 52.5 degrees of the pulp cavity
	Score 3—involved into the 52.5 to 60 degrees of the pulp cavity
	Score 4—involved into the 60 to 67.5 degrees of the pulp cavity
	Score 5—involved into the 67.5 to 75 degrees of the pulp cavity
	Score 6—involved into the 75 to 82.5 degrees of the pulp cavity
	Score 7—involved into the 82.5 to 90 degrees of the pulp cavity
	Score 8—involved into the 90 to 97.5 degrees of the pulp cavity
	Score 9—involved into the 97.5 to 105 degrees of the pulp cavity
Intensity of the inflammatory infiltrate	Score 1—0 to 20 inflammatory cells
	Score 2—20 to 40 inflammatory cells
	Score 3—40 to 60 inflammatory cells
	Score 4—over 60 inflammatory cells
Inflammatory edema	Score 1—absent
	Score 2—present
Extension of pulp necrosis (45 to 105 degrees area)	Score 1—absent
	Score 2—1%-20% necrotic area
	Score 3—20%-30% necrotic area
	Score 4—over 30% necrotic area

**Table 2:** Histomorphologic scores per group (%).

Parameters	Scores	Control (*n* = 8)	AP (*n* = 8)	AP+melatonin (*n* = 8)	AP+5-MTX (*n* = 8)
Locations of the inflammatory reaction	1	100	0	0	0
	2	0	0	0	0
	3	0	0	12.5	0
	4	0	0	37.5	50
	5	0	87.5	50	50
	6	0	12.5	0	0
	7	0	0	0	0
	8	0	0	0	0
	9	0	0	0	0
Intensity of the inflammatory infiltrate	1	100	0	0	0
	2	0	0	0	12.5
	3	0	0	50	50
	4	0	100	50	37.5
Inflammatory edema	1	100	0	0	0
	2	0	100	100	100
Extension of pulp necrosis (45 to 105 degrees area)	1	100	0	0	0
	2	0	0	0	0
	3	0	62.5	75	87.5
	4	0	37.5	25	12.5

## Data Availability

Data used to support the findings of this study can be obtained from the relevant author upon request.

## References

[1] Mjör I. A., Sveen O. B., Heyeraas K. J. (2001). Pulp-dentin biology in restorative dentistry. Part 1: normal structure and physiology. *Quintessence International*.

[2] Zero D. T., Zandona A. F., Vail M. M., Spolnik K. J. (2011). Dental caries and pulpal disease. *Dental Clinics of North America*.

[3] Hehlgans T., Pfeffer K. (2005). The intriguing biology of the tumour necrosis factor/tumour necrosis factor receptor superfamily: players, rules and the games. *Immunology*.

[4] Chang M. C., Lin L. D., Zwei-Ching Chang J. (2012). Regulation of vascular cell adhesion molecule-1 in dental pulp cells by interleukin-1*β*: the role of prostanoids. *Journal of Endodontics*.

[5] Zheng L., Amano K., Iohara K. (2009). Matrix metalloproteinase-3 accelerates wound healing following dental pulp injury. *American Journal of Pathology*.

[6] Jain A., Bahuguna R. (2015). Role of matrix metalloproteinases in dental caries, pulp and periapical inflammation: an overview. *Journal of Oral Biology and Craniofacial Research*.

[7] Shin S.-J., Lee J.-I., Baek S.-H., Lim S.-S. (2002). Tissue levels of matrix metalloproteinases in pulps and periapical lesions. *Journal of Endodontics*.

[8] Wahlgren J., Salo T., Teronen O., Luoto H., Sorsa T., Tjäderhane L. (2002). Matrix metalloproteinase-8 [MMP-8] in pulpal and periapical inflammation and periapical root-canal exudates. *International Endodontic Journal*.

[9] Rudra D. S., Pal U., Maiti N. C., Reiter R. J., Swarnakar S. (2013). Melatonin inhibits matrix metalloproteinase-9 activity by binding to its active site. *Journal of Pineal Research*.

[10] Esposito E., Genovese T., Caminiti R., Bramanti P., Meli R., Cuzzocrea S. (2008). Melatonin regulates matrix metalloproteinases after traumatic experimental spinal cord injury. *Journal of Pineal Research*.

[11] Esposito E., Mazzon E., Riccardi L., Caminiti R., Meli R., Cuzzocrea S. (2008). Matrix metalloproteinase-9 and metalloproteinase-2 activity and expression is reduced by melatonin during experimental colitis. *Journal of Pineal Research*.

[12] Kara A., Akman S., Ozkanlar S. (2013). Immune modulatory and antioxidant effects of melatonin in experimental periodontitis in rats. *Free Radical Biology and Medicine*.

[13] Li J. G., Lin J. J., Wang Z. L. (2015). Melatonin attenuates inflammation of acute pulpitis subjected to dental pulp injury. *American Journal of Translational Research*.

[14] Satué M., Ramis J. M., Del Mar Arriero M., Monjo M. (2015). A new role for 5-methoxytryptophol on bone cells function in vitro. *Journal of Cellular Biochemistry*.

[15] Ouzir M., Bouhaddou N., Khalki H., Lakhdar-Ghazal N. (2014). Physiological and pharmacological properties of 5-methoxytryptophol. *Expert Review of Endocrinology & Metabolism*.

[16] Savtekin G., Tuzum M. S., Uyanik L. O. (2016). Effects of melatonin and 5-methoxytryptophol on synovial inflammation in the zymosan-induced rheumatoid arthritis in rats. *International Journal of Clinical and Experimental Medicine*.

[17] Akman S., Canakci V., Kara A., Tozoglu U., Arabaci T., Dagsuyu İ. M. (2013). Therapeutic effects of alpha-lipoic acid and vitamin C on alveolar bone resorption after experimental periodontitis in rats. A biochemical, histochemical and stereologic study. *Journal of Periodontology*.

[18] Aksoy U., Savtekin G., Şehirli A. Ö. (2019). Effects of alpha-lipoic acid therapy on experimentally induced apical periodontitis: a biochemical, histopathological and micro-CT analysis. *International Endodontic Journal*.

[19] Azuma M. M., Gomes-Filho J. E., Cardoso C. D. (2018). Omega 3 fatty acids reduce the triglyceride levels in rats with apical periodontitis. *Brazilian Dental Journal*.

[20] Brilhante Wolle C. F., De Aguiar Zollmann L., Etges A., Vitalis G. S., Leite C. E., Campos M. M. (2012). Effects of the antioxidant agent tempol on periapical lesions in rats with doxorubicin-induced cardiomyopathy. *Journal of Endodontics*.

[21] Renn T. Y., Huang Y. K., Feng S. W. (2018). Prophylactic supplement with melatonin successfully suppresses the pathogenesis of periodontitis through normalizing RANKL/OPG ratio and depressing the TLR4/MyD88 signaling pathway. *Journal of Pineal Research*.

[22] Virto L., Haugen H. J., Fernández-Mateos P. (2018). Melatonin expression in periodontitis and obesity: an experimental in-vivo investigation. *Journal of Periodontal Research*.

[23] Wolle C. F. B., Zollmann L. A., Bairros P. O. (2013). Outcome of periapical lesions in a rat model of type 2 diabetes: refractoriness to systemic antioxidant therapy. *Journal of Endodontics*.

[24] Elsalhy M., Azizieh F., Raghupathy R. (2013). Cytokines as diagnostic markers of pulpal inflammation. *International Endodontic Journal*.

[25] Bradford M. M. (1976). A rapid and sensitive method for the quantitation of microgram quantities of protein utilizing the principle of protein-dye binding. *Analytical Biochemistry*.

[26] Cutando A., Montero J., Gómez-de Diego R., Ferrera M. J., Lopez-Valverde A. (2015). Effect of topical application of melatonin on serum levels of C-reactive protein (CRP), interleukin-6 (IL-6) and tumor necrosis factor-alpha (TNF-*α*) in patients with type 1 or type 2 diabetes and periodontal disease. *Journal of Clinical and Experimental Dentistry*.

[27] Chung M.-K., Lee J., Duraes G., Ro J. Y. (2011). Lipopolysaccharide-induced pulpitis up-regulates TRPV1 in trigeminal ganglia. *Journal of Dental Research*.

[28] Cleaton-Jones P., Duggal M., Parak R., Williams S., Setzer S. (2004). Pulpitis induction in baboon primary teeth using carious dentine or Streptococcus mutans. *Sadj*.

[29] Lin J. J., Du Y., Cai W. K. (2015). Toll-like receptor 4 signaling in neurons of trigeminal ganglion contributes to nociception induced by acute pulpitis in rats. *Scientific Reports*.

[30] Ma J., Chen W., Zhang L. (2013). RNA interference-mediated silencing of Atp6i prevents both periapical bone erosion and inflammation in the mouse model of endodontic disease. *Infection and Immunity*.

[31] Kawanishi H. N., Kawashima N., Suzuki N., Suda H., Takagi M. (2004). Effects of an inducible nitric oxide synthase inhibitor on experimentally induced rat pulpitis. *European Journal of Oral Sciences*.

[32] Kawashima N., Nakano-Kawanishi H., Suzuki N., Takagi M., Suda H. (2005). Effect of NOS inhibitor on cytokine and COX2 expression in rat pulpitis. *Practitioner*.

[33] Rupf S., Kannengießer S., Merte K., Pfister W., Sigusch B., Eschrich K. (2000). Comparison of profiles of key periodontal pathogens in periodontium and endodontium. *Dental Traumatology*.

[34] Tarsa L., Bałkowiec-Iskra E., Kratochvil F. J. (2010). Tooth pulp inflammation increases brain-derived neurotrophic factor expression in rodent trigeminal ganglion neurons. *Neuroscience*.

[35] Hsieh S. C., Tsao J. T., Lew W. Z. (2015). Static magnetic field attenuates lipopolysaccharide-induced inflammation in pulp cells by affecting cell membrane stability. *Scientific World Journal*.

[36] Lee J.-C., Yu M.-K., Lee R. (2008). Terrein reduces pulpal inflammation in human dental pulp cells. *Journal of Endodontics*.

[37] Yu M.-K., Lee J.-C., Kim J.-H. (2009). Anti-inflammatory effect of peroxisome proliferator activated receptor gamma on human dental pulp cells. *Journal of Endodontics*.

[38] Renard E., Gaudin A., Bienvenu G. (2016). Immune cells and molecular networks in experimentally induced pulpitis. *Journal of Dental Research*.

[39] Huang J., Lv Y., Fu Y. (2015). Dynamic regulation of delta-opioid receptor in rat trigeminal ganglion neurons by lipopolysaccharide-induced acute pulpitis. *Journal of Endodontics*.

[40] He Y., Gan Y., Lu J. (2016). Pulpal tissue inflammatory reactions after experimental pulpal exposure in mice. *Journal of Endodontics*.

[41] Gery I., Gershon R. K., Waksman B. H. (1972). Potentiation of the T-lymphocyte response to mitogens. I. The responding cell. *The Journal of Experimental Medicine*.

[42] Rhim E.-M., Park S.-H., Kim D.-S., Kim S.-Y., Choi K.-K., Choi G.-W. (2011). The effect of tumor necrosis factor [TNF]-*α* to induce matrix metalloproteinase [MMPs] from the human dental pulp, gingival, and periodontal ligament cells. *Journal of Korean Academy of Conservative Dentistry*.

[43] Gómez-Moreno G., Cutando-Soriano A., Arana C. (2007). Melatonin expression in periodontal disease. *Journal of Periodontal Research*.

[44] Srinath R., Acharya A. B., Thakur S. L. (2010). Salivary and gingival crevicular fluid melatonin in periodontal health and disease. *Journal of Periodontology*.

[45] Lissoni P., Pittalis S., Rovelli F. (1996). Immunomodulatory properties of a pineal indole hormone other than melatonin, the 5-methoxytryptophol. *Journal of Biological Regulators and Homeostatic Agents*.

[46] Chen Q., Jin M., Yang F., Zhu J., Xiao Q., Zhang L. (2013). Matrix metalloproteinases: inflammatory regulators of cell behaviors in vascular formation and remodeling. *Mediators of Inflammation*.

[47] Xue M., March L., Sambrook P. N., Jackson C. J. (2007). Differential regulation of matrix metalloproteinase 2 and matrix metalloproteinase 9 by activated protein C: relevance to inflammation in rheumatoid arthritis. *Arthritis and Rheumatism*.

[48] Clegg P. D., Burke R. M., Coughlan A. R., Riggs C. M., Carter S. D. (1997). Characterisation of equine matrix metalloproteinase 2 and 9; and identification of the cellular sources of these enzymes in joints. *Equine Veterinary Journal*.

[49] Cunnane G., Fitzgerald O., Hummel K. M. (2001). Synovial tissue protease gene expression and joint erosions in early rheumatoid arthritis. *Arthritis and Rheumatism*.

